# Perceiving the Concealed and Unreported Pharmacophoric Features of the 5-Hydroxytryptamine Receptor Using Balanced QSAR Analysis

**DOI:** 10.3390/ph15070834

**Published:** 2022-07-05

**Authors:** Syed Nasir Abbas Bukhari, Mervat Abdelaziz Elsherif, Kashaf Junaid, Hasan Ejaz, Pravej Alam, Abdul Samad, Rahul D. Jawarkar, Vijay H. Masand

**Affiliations:** 1 Department of Pharmaceutical Chemistry, College of Pharmacy, Jouf University, Sakaka 72388, Saudi Arabia; 2 Chemistry Department, College of Science, Jouf University, Sakaka 72388, Saudi Arabia; maelsherif@ju.edu.sa; 3 Department of Clinical Laboratory Sciences, College of Applied Medical Sciences, Jouf University, Sakaka 72388, Saudi Arabia; kjunaid@ju.edu.sa (K.J.); hetariq@ju.edu.sa (H.E.); 4 Department of Biology, College of Science and Humanities, Prince Sattam Bin Abdulaziz University, Al-Kharj 11942, Saudi Arabia; alamprez@gmail.com; 5 Department of Pharmaceutical Chemistry, Faculty of Pharmacy, Tishk International University, Erbil 44001, Iraq; abdul.samad@tiu.edu.iq; 6 Department of Medicinal Chemistry, Dr. Rajendra Gode Institute of Pharmacy, University-Mardi Road, Amravati 444603, Maharashtra, India; rahuljawarkar@gmail.com; 7 Department of Chemistry, Vidya Bharati Mahavidyalaya, Amravati 444602, Maharashtra, India

**Keywords:** 5-hydroxytryptamine receptor 6, neurodegeneration, QSAR, molecular docking, pharmacophoric features

## Abstract

The 5-hydroxytryptamine receptor 6 (5-HT6) has gained attention as a target for developing therapeutics for Alzheimer’s disease, schizophrenia, cognitive dysfunctions, anxiety, and depression, to list a few. In the present analysis, a larger and diverse dataset of 1278 molecules covering a broad chemical and activity space was used to identify visual and concealed structural features associated with binding affinity for 5-HT6. For this, quantitative structure–activity relationships (QSAR) and molecular docking analyses were executed. This led to the development of a statistically robust QSAR model with a balance of excellent predictivity (R^2^_tr_ = 0.78, R^2^_ex_ = 0.77), the identification of unreported aspects of known features, and also novel mechanistic interpretations. Molecular docking and QSAR provided similar as well as complementary results. The present analysis indicates that the partial charges on ring carbons present within four bonds from a sulfur atom, the occurrence of sp3-hybridized carbon atoms bonded with donor atoms, and a conditional occurrence of lipophilic atoms/groups from nitrogen atoms, which are prominent but unreported pharmacophores that should be considered while optimizing a molecule for 5-HT6. Thus, the present analysis led to identification of some novel unreported structural features that govern the binding affinity of a molecule. The results could be beneficial in optimizing the molecules for 5-HT6.

## 1. Introduction

**Figure 1:**
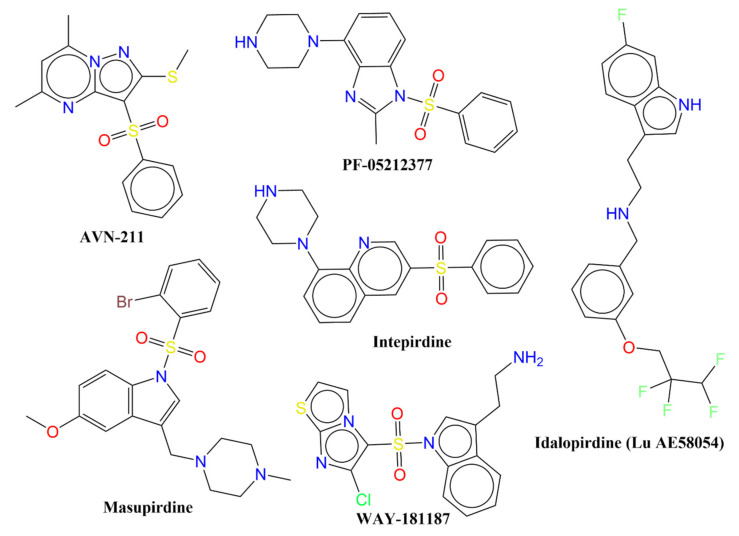
Chemical structures of representative examples of clinically tested 5-HT6 ligands.

Millions of people are suffering from central nervous system (CNS)-related diseases, such as Alzheimer’s disease, schizophrenia, cognitive dysfunctions, anxiety, depression, etc. [1,2,3,4], and are facing diverse health and social challenges. Though therapeutic agents are available for the treatment of these diseases, they lack the ability to reduce the continuous loss of cognitive function, as most of them target only the acetylcholine deficit [2,3,5,6,7,8,9]. Therefore, there is a need to develop a therapeutic agent with a different mechanism of action and bio-activity profile to support the existing drug space. Recently, G-protein-coupled receptors (GPCRs), viz., the serotonergic system of serotonin (5-hydroxytryptamine) receptors, have received greater attention due to their vital role in signal transduction pathways and numerous neurological functions [2,4,7,8,9,10]. To add further, 5-hydroxytryptamine receptors (5-HT_1–7_) play vital roles in many cognitive dysfunctions such as memory loss, reduced learning ability, etc. [4,5,8,9]. Among them, the 5-HT6 receptor has emerged as a promising target due to its crucial role in the onset of Alzheimer’s disease, cognitive processes, mood control, depression, and anxiety [4,5,8,9], to name a few. It is believed that blocking the 5-HT6 receptor significantly improves learning and memory processes [4,5,8,9]. In addition, it has also emerged as a molecular target for the treatment of obesity and the related metabolic syndrome [11]. An additional advantage associated with 5-HT6 is its restricted and exclusive occurrence within the CNS, which implies that compounds acting through this receptor could have minimal peripheral side effects [2,3,6,10,12]. Even though several molecules have been identified as promising ligands with high binding affinities for 5-HT6 (see Figure 1), none of them has cleared the clinical stages or been approved as a drug [1,7,8,9,10,12].

Therefore, there is a need to develop a novel therapeutic agent with a better ADMET (absorption, distribution, metabolism, excretion, and toxicity) profile and the retention of a high binding affinity for 5-HT6. For this, it is essential to know the prominent and concealed pharmacophoric features associated with binding affinities for 5-HT6 using a broad, structurally diverse dataset with adequate variations in activity space. To achieve these targets, computer-aided drug designing (CADD) is a contemporary and feasible solution due to its low cost and time efficacy [13,14]. Ligand-based drug designing (LBDD) is a thriving and widely accepted branch of CADD with a higher success rate of identification of key pharmacophoric features. It is the method of choice if the 3D structure of a target protein is not available [14]. Under the terrain of LBDD, the quantitative structure–activity relationship (QSAR) is an effective and multidisciplinary approach that is useful to identify salient and hidden pharmacophoric features [15,16,17,18].

Though different types of ligands encompassing diverse scaffolds are known for 5-HT6, the lack of an X-ray-resolved 3D structure restricts the use of structure-based drug designing (SBDD) approaches. Consequently, many researchers have performed QSAR analysis for 5-HT6 using different types of scaffolds. Doddareddy and co-workers [1] accomplished a 3D QSAR analysis using a small dataset of 33 N1-arylsulfonylindole compounds as 5-HT6 antagonists. The analysis reinforced the pharmacophoric features reported by López-Rodríguez et al. [4,19]. Later in 2011, Hao et al. [3] studied relatively a larger dataset of 223 ligands reported for 5-HT6 for QSAR, homology-based molecular docking, and molecular dynamics simulations for 5 ns. Their analysis pointed out that the interaction with the residue Asp106 is important. A 2D and 3D QSAR analysis of arylsulfonamide-derived 5-HT6 receptor antagonists [12] highlighted the importance of the pharmacophore model reported by López-Rodríguez et al. [4,19]. Though these studies contributed to the identification of some important structural features, the developed QSAR models were either based on smaller datasets or specific scaffolds only, thereby lacking general applicability. In addition, poor external predictive ability restricts their usage for a lead optimization pipeline. A QSAR analysis based on a large and diverse set of molecules, with a balance of predictive ability (predictive QSAR) and mechanistic interpretation (mechanistic QSAR) provides an in-depth understanding of the correlation between the structural features and the desired bio-activity [15,20,21]. Therefore, QSAR analysis has been executed in the present work using a large and diverse dataset that covers a broad chemical and activity space to find out the structural features of high importance for 5-HT6 ligands. Molecular docking has been carried out to support and complement the QSAR analysis. Further, an in-depth analysis of a larger dataset comprising diverse scaffolds led to the identification of reported as well as unreported pharmacophoric features, which could be useful in the optimization of molecules during a drug discovery pipeline. 

## 2. Results

In the present work, we have identified reported as well as unreported structural features of 5-HT6 ligands. As stated in the introduction section, the emphasis was on building a genetic algorithm–multilinear regression (GA-MLR) model with a balance of predictive ability and mechanistic interpretations. The newly built six-parametric model is as follows:

**Model-1:** pKi = 6.754 (±0.091) −0.109 (±0.014) * com_Hhyd_3A −0.700 (±0.043) * ringC_S_4Bc −0.604 (±0.104) * flipo&S_ringN3B −0.528 (±0.075) * KRFPC620 −0.339 (±0.053) * sp3N_sp2O_8B + 0.545 (±0.067) * fsp3Cdon1B

The details of the molecular descriptors present in Model-1 have been tabulated in Table 1 and are discussed in detail in the discussion section. Table 2 contains selected validation parameters associated with Model-1.

It is evident from above statistical parameters that the model is statistically predictive, with high values of different parameters such as R^2^_tr_ (coefficient of determination), R^2^_adj._ (adjusted coefficient of determination), and R^2^_cv_ (Q^2^_LOO_) (cross-validated coefficient of determination for leave-one-out), R^2^_ex_ (external coefficient of determination), Q^2^−F^n^, and CCC_ex_ (concordance correlation coefficient for external set), etc., and low values of LOF (lack-of-fit), RMSE (root-mean-square error), MAE (mean absolute error), R^2^_Yscr_ (R^2^ for Y-scrambling), etc. Thus, the model possesses high external predictive ability, is free from chance correlations, and satisfies the recommended threshold values for various validation parameters [16,22,23,24,25,26,27,28,29]. All validation parameters associated with Model-1 and their formulae are available in the Supplementary Materials.

**Figure 2:**
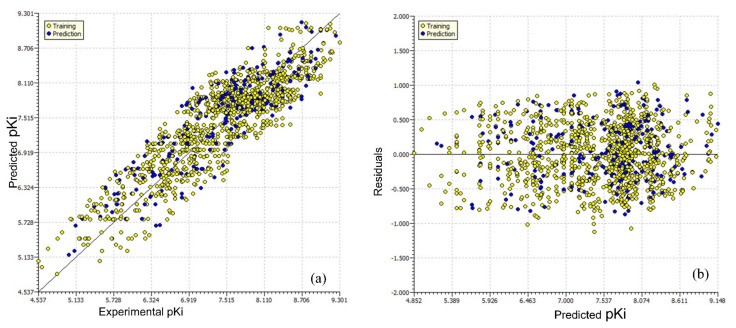

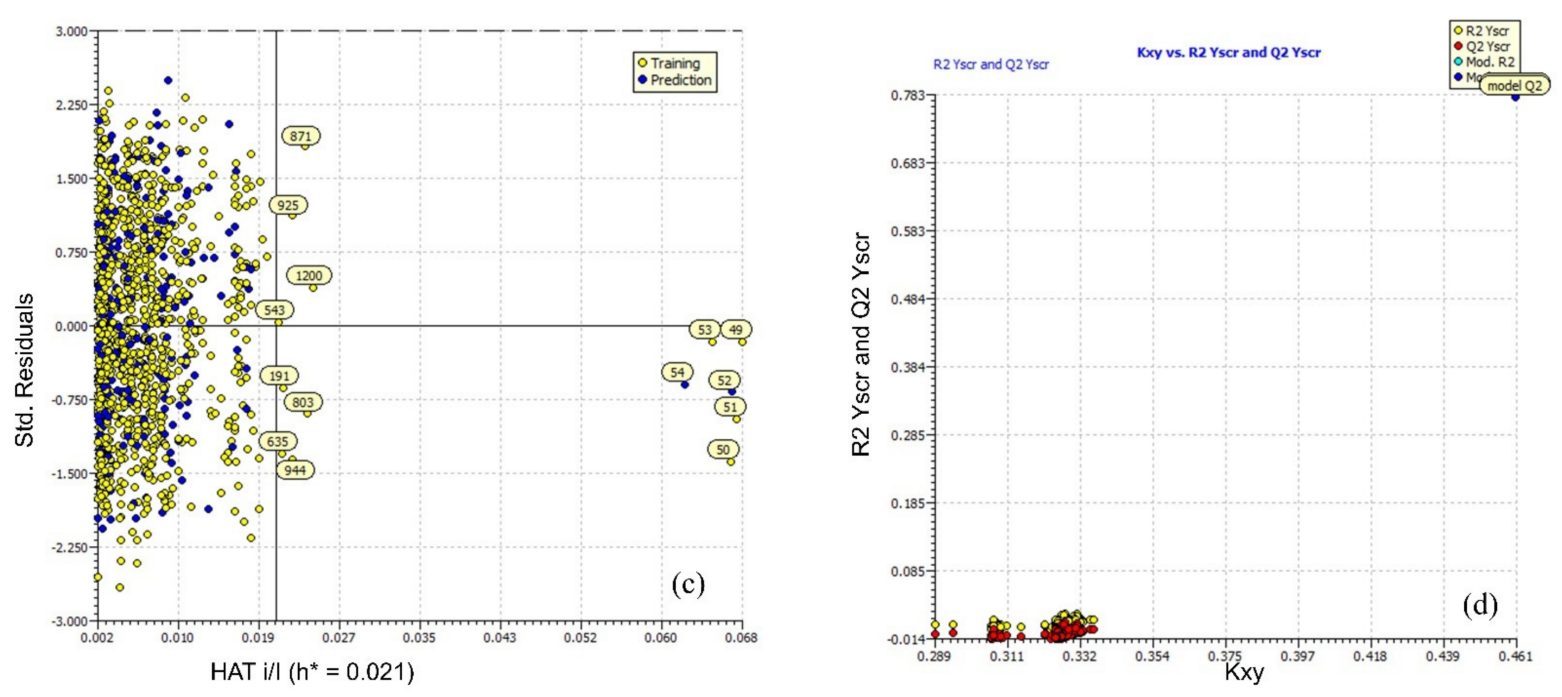
Different graphs associated with the Model-1. (**a**) Experimental vs. predicted pKi (the solid line represents the regression line); (**b**) experimental vs. residuals; (**c**) Williams plot for applicability domain (the vertical solid line represents h* = 0.021 and the horizontal dashed lines represent the upper and lower boundaries of the applicability domain); (**d**) Y-randomization plot.

Figure 2 contains the different graphs associated with Model-1, viz., the experimental vs. predicted pKi (Figure 2a), experimental vs. residuals (Figure 2b), and the Y-randomization plot (Figure 2d). We used a Williams plot to judge the applicability domain of the model (see Figure 2c). Thus, it satisfies all the OECD-endorsed guidelines and criteria for creating a useful QSAR model.

The docking scores for all the molecules are incorporated in the Supplementary Materials (see Excel file ‘SupplementaryMaterial-Final.xlsx’, available in the Supplementary Materials).

## 3. Discussion

The interpretation of a QSAR model using molecular descriptors, also known as mechanistic interpretation, is a crucial aspect to highlight and link various structural features with significant influence in deciding the bio-activity of molecules [15,21]. It is also one of the requirements suggested by the OECD for developing a thriving QSAR model. In the present work, we used a simple approach in which the pKi values of different molecules were compared using a specific molecular descriptor. Nonetheless, we make clear that the final experimental pKi value of a molecule cannot be governed by just a single structural feature (molecular descriptor). To add further, the extending or reverse effect of other molecular descriptors and unknown factors play crucial roles in deciding the pKi value of a molecule. Therefore, a concomitant consideration of all molecular descriptors and their associated structural features is a better strategy for the effective use of a QSAR model. Model-1 encompasses six molecular descriptors.

**Figure 3:**
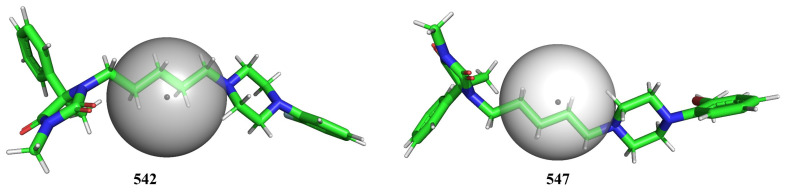
Depiction of com_Hhyd_3A using molecules 542 and 547 as representative examples (radius of gray sphere is 3 Å).

The molecular descriptor com_Hhyd_3A represents the total number of hydrogen atoms with a partial charge in the range of ±0.200 and also present within 3 Å from the center of mass (com) of the molecule. As the partial charge must be within the range ±0.200, this descriptor expresses the role played by non-polar hydrogens [30] when present within 3Å from the center of mass of the molecule. The descriptor com_Hhyd_3A has a negative coefficient in Model-1, which indicates that the lower the value, the better the binding affinity. This could be achieved by replacing non-polar hydrogen atoms with suitable atoms/groups. This indirectly points out that the presence of polar hydrogens, in turn, polar groups nearer to center of mass of a molecule, are beneficial for escalating the pKi value. In addition, hydrogen is smaller than other elements, and replacing it with any other element will increase the steric bulk. Therefore, bulkiness near the center of mass of the molecule is highly favorable for increasing the pKi value. To add further, com_Hhyd_3A depends on the location of the center of mass, which changes with the positions of groups/atoms (positional isomers). Therefore, the value of com_Hhyd_3A varies for positional isomers, for example, molecules number 331 and 332. Hence, the descriptor effortlessly captures the importance of positional isomerism in deciding the pKi value. Thus, the descriptor com_Hhyd_3A and its negative correlation (correlation coefficient R = −0.63) with pKi highlighted the crucial role played by the presence of polar groups and steric bulkiness near the center of mass of a molecule as well as the positional isomerism. This observation was further confirmed by comparing following pairs of molecules: 542 (Ki = 25520 nM, com_Hhyd_3A = 8) with 547 (Ki = 2506 nM, com_Hhyd_3A = 6) (depicted in Figure 3), 543 (Ki = 14650 nM, com_Hhyd_3A = 9) with 545 (Ki = 8611 nM, com_Hhyd_3A = 6), 1011 (Ki = 91 nM, com_Hhyd_3A = 6) with 1082 (Ki = 419 nM, com_Hhyd_3A = 7), 935 (Ki = 2843 nM, com_Hhyd_3A = 8) vs. 937 (Ki = 2005 nM, com_Hhyd_3A = 6), and 929 (Ki = 2427 nM, com_Hhyd_3A = 7) vs. 938 (Ki = 1540 nM, com_Hhyd_3A = 5), to list a few. Thus, for the first time, com has been used as a useful approach to explain the differences in the binding affinities of ligands for 5-HT6. In addition, this novel approach provides a novel justification for the differences in the activity of positional isomers.

**Figure 4:**
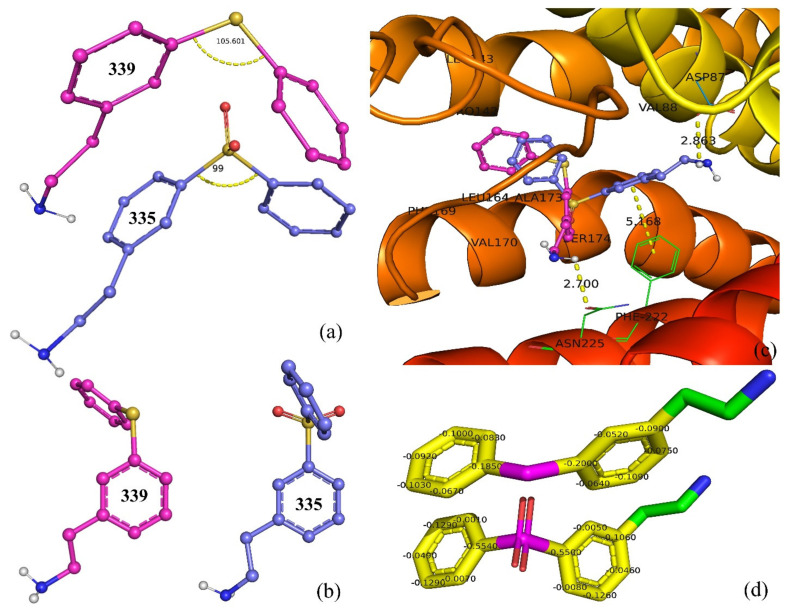
Molecules 335 and 339 as representative examples to depict the effect of descriptor ringC_S_4Bc. (**a**) Comparison of docking pose of 335 (pink) and 339 (blue), (**b**) C-S-C bond angle shown using yellow dotted lines, (**c**) docking pose for 335 (pink) and 339 (blue), (**d**) carbon atoms (yellow) within four bonds of sulfur (magenta) with respective partial charges.

ringC_S_4Bc, which signifies the sum of partial charges on ring carbon atoms present within four bonds from a sulfur atom, has a negative coefficient in Model-1. In addition, it has a correlation coefficient of −0.696 with pKi. Therefore, decreasing the value of ringC_S_4Bc could lead to a higher pKi value, i.e., a better binding affinity. The descriptor highlights the importance of ring carbon and sulfur atoms; therefore, it looks as if merely ring carbon or sulfur atoms are enough to control the binding affinity. Replacing it with nringC (the total number of ring carbon atoms) or nS (the total number of sulfur atoms) reduced the statistical performance of Model-1 (R^2^ = 0.74 and 0.73). Therefore, a combined presence of ring carbons and sulfur atoms within four bonds from each other has more influence. The descriptor is shown using molecules 335 and 339 as representative examples in Figure 4. A comparison of the following pairs of molecules confirmed the observation: 335 with 339, 952 with 953, 954 with 955, 248 with 249, 762 with 763, 221 with 260, 175 with 650, 1089 with 1101, 989 with 1030, etc.

A literature survey revealed that a combination of a H-bond acceptor, such as a sulfonamide or sulfone moiety, along with fused rings such as naphthalene, benzothiophene, or indole, is highly suitable for augmenting the pKi values of 5-HT6 ligands [6] due to their hydrophobic character [5]. Interestingly, the sulfone or sulfonamide groups are directly attached to fused rings in many compounds of the present dataset. In addition, in the present work, we found that the partial charges on the ring carbons of fused rings also influenced the binding affinity of a molecule for 5-HT6. Therefore, the hydrophobic character of fused rings and the partial charges on a ring carbon are equally important. This observation is further supported by the molecular docking analysis. The molecular docking analysis revealed that molecules number 335 and 339 have different poses and types of interactions. Molecule 335 has an established H-bond with Asp87 (distance 2.86 Å) and a pi–pi interaction with Phe-222 (distance 5.17 Å), whereas molecule 339 has only a H-bond formation with Asn225 (distance 2.70 Å) and lacks any pi–pi interactions. Molecule 335 was able to establish a pi–pi interaction due to the presence of a sulfone group, which caused substantial changes in the partial charges of ring carbon atoms and the C-S-C bond angle (99.8° in 335 and 105.6° in 339) and also changed the solvent-accessible surface areas (2312.8 Å^2^ for 335 and 2098.4 Å^2^ for 339). The docking poses for molecules number 335 and 339 are shown in Figure 4c. The yellow dotted line represents the prominent interaction with distances in Angstrom units. It is clear from the present analysis that not only lipophilic factors but electronic factors associated with ring carbon atoms vicinal to sulfur, in turn aromatic/aliphatic rings, are an important parameter in deciding the inhibitory activity for 5-HT6. Thus, the present work is successful in identifying novel unreported crucial aspects of a previously known pharmacophoric feature required for better activity for 5-HT6.

**Figure 5:**
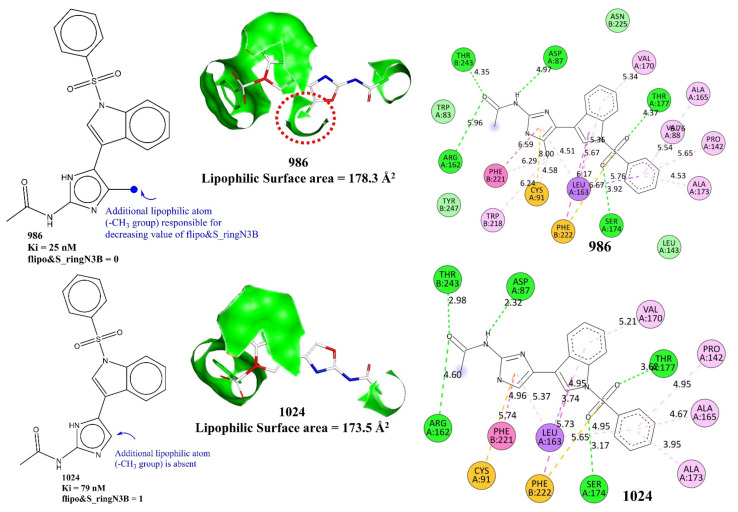
Representation of flipo&S_ringN3B using molecules 986 and 1024 as examples. The green contour in the middle figures depicts the lipophilic surface area.

The molecular descriptor flipo&S_ringN3B represents the frequency of occurrence of a ring nitrogen atom exactly three bonds from a lipophilic atom or sulfur atom. If the same ring nitrogen atom is also present at two or less bonds from any other lipophilic atom or sulfur atom, then it is neglected while calculating flipo&S_ringN3B. It has a negative coefficient in Model-1, which suggests that lowering the value of flipo&S_ringN3B could lead to a better binding profile. This observation is justified by the fact that there are 383 molecules with Ki ≤ 10 nM, but only one of them has flipo&S_ringN3B = 2 (molecule number 50). Only two molecules (molecules number 569 and 978) possess flipo&S_ringN3B = 1, and the remaining 380 molecules have flipo&S_ringN3B = 0. A comparison of molecules 870 with 871, 896 with 903, and 892 with 897 and 898 further strengthens the observation. For example, consider molecules 986 and 1024. In the case of molecule 986, the presence of an additional -CH_3_ group on the imidazole ring increased the number of lipophilic atoms within three bonds of the ring nitrogen as well as the lipophilic surface area (178.3 Å^2^), thereby having flipo&S_ringN3B = 0, while molecule 1024 lacks such a substituent and consequently has flipo&S_ringN3B = 1 and a lower lipophilic surface area of 173.5 Å^2^ (See Figure 5). 

Likewise, in the case of highly active molecules, a lower value of flipo&S_ringN3B is possible due to a lower number of ring nitrogen (for example molecule 681) or due to the presence of lipophilic atoms within three bonds of a ring nitrogen. Thus, this descriptor signifies that the presence of lipophilic atoms near ring nitrogen atoms decreases the value of flipo&S_ringN3B, which in turn increases the pKi value. In other words, it points out an unreported structural feature of conditional importance of lipophilic atoms as well as ring nitrogen atoms in deciding the binding affinity profile. A plausible reason could be attributed to the fact that increasing the lipophilic environment around a ring nitrogen balances the polarity induced by the highly electronegative nature of nitrogen, which ultimately leads to improved brain penetration [4] due to the better lipophilic characters of the molecule. The molecular docking poses for molecules number 986 and 1024 (see Figure 5) indicate that the additional -CH_3_ group is responsible for pi-alkyl hydrophobic interactions with the active site residues of 5-HT6 for molecule number 986. In the case of molecule number 986, the additional -CH_3_ group interacts with Phe221, Cys91, and Trp218. The interaction with Trp218 is absent in case of molecule number 1024. 

**Figure 6:**
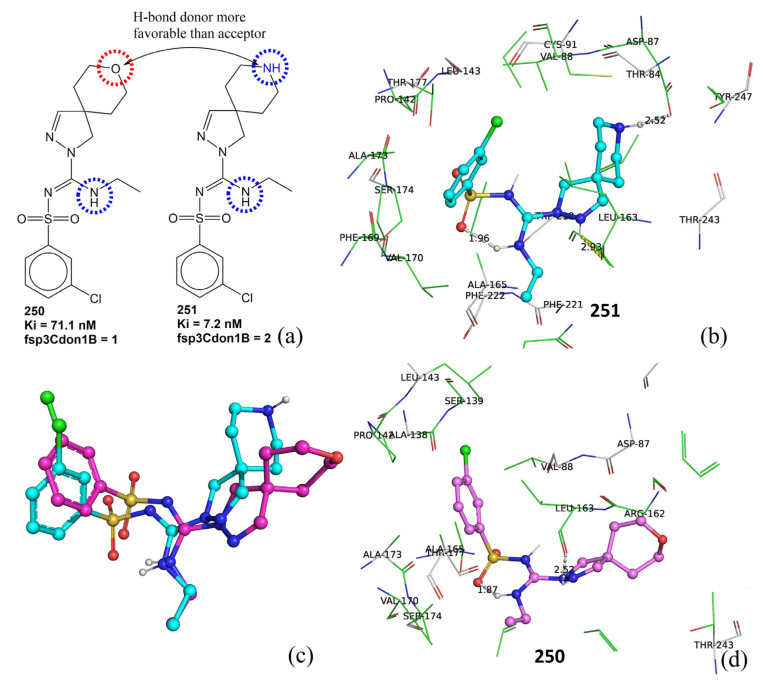
(**a**) Representation of fsp3Cdon1B using molecules number 250 and 251 as examples, (**b**) docking pose for molecule number 251 (dotted line represents H-bond formation), (**c**) overlap of docking pose of 250 (cyan) and 251 (pink), (**d**) docking pose for 250.

The molecular descriptor fsp3Cdon1B, which signifies the frequency of occurrence of a H-bond donor atom directly bonded with an sp^3^-hybridized carbon atom, has a positive coefficient in Model-1. Therefore, increasing such a combination of carbon and H-bond donor atoms could lead to a higher pKi value. An inspection of molecules with Ki ≤ 10 nM (383 molecules) reveals that only 16 molecules lack such a combination of donor and carbon, 286 molecules have one such combination, and 82 molecules possess fsp3Cdon1B = 2. A comparison of molecules number 250 (Ki = 71.1 nM, fsp3Cdon1B = 0) and 251 (Ki = 7.2 nM, fsp3Cdon1B = 1) further supports the positive effect of the presence of fsp3Cdon1B on the pKi value (see Figure 6). The molecular docking poses for molecules number 250 and 251 support this observation (see Figure 6b–d). Both molecules interact with similar residues such as Leu163 (H-bond). However, a prominent difference in the docking poses of molecules number 251 and 250 is the H-bond formation with a distance of 2.52 Å by the -NH of the piperidine ring with Asp87, which is absent in the case of 250. This is due to the fact that the pyran ring of molecule 250 is oriented away from Asp87, whereas the piperidine ring of 251 is close enough to establish a H-bond with Asp87. Thus, it highlights the importance of the presence of a donor atom in the 5-HT6 ligand. The -NH of the piperidine ring is responsible for fsp3Cdon1B = 1 for molecule number 250. Thus, the molecular docking and QSAR provided similar and complementary results.

**Figure 7:**
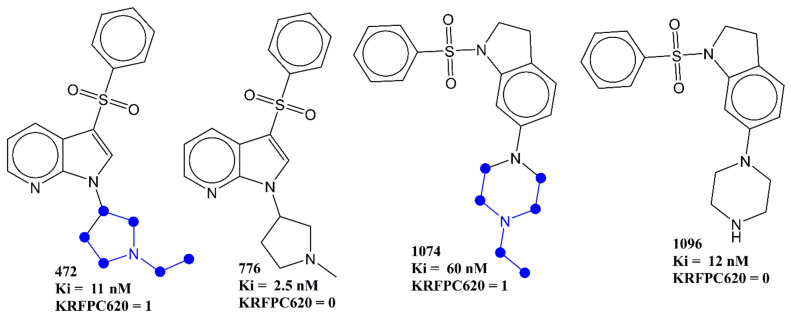
Pictorial representation of KRFPC660 (blue colored) using representative examples.

Another molecular descriptor which highlights the importance of nitrogen atoms and their local environment is KRFPC620. The molecular descriptor KRFPC620 considers the count of a fragment that encompasses a planer nitrogen atom attached to three CH_3_-CH_2_- groups. For better clarification, it is depicted in Figure 7 using representative examples only. The negative coefficient for the descriptor indicates that the pKi value increases with a decrease in the value of KRFPC620. A comparative analysis of the following pairs of molecules supports this trend: 472 with 776, 748 with 749, 757 with 761, 1074 with 1096, 1068 with 1075, 1100 with 1116, 1094 with 1058, etc. To add further, of the 383 molecules with Ki ≤ 10 nM, 37 molecules have KRFPC620 = 1, whereas 346 molecules lack the presence of KRFPC620. Therefore, such a combination of nitrogen with CH_3_-CH_2_- must be avoided for a better pKi value.

Many researchers have highlighted the importance of the presence of a positive ionizable atom or group such as the nitrogen in piperazine or in a (dimethylamino)ethyl fragment for a better binding affinity for 5-HT6 [4]. The present work also successfully highlighted the negative impact of a third ethyl group on ionizable nitrogen on binding affinity. Therefore, in agreement with previous studies [4,12], a methyl substituted piperazine, non-substituted piperazine, or a (dimethylamino)ethyl fragment is a better choice to act as a positive ionizable moiety. 

**Figure 8:**
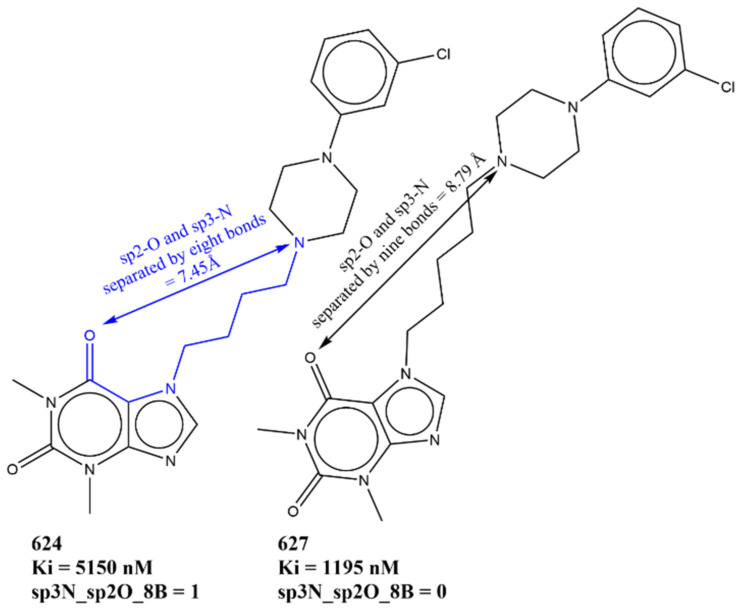
Illustrative examples for representation of sp3N_sp2O_8B.

Another descriptor whose value must be lowered due to its negative coefficient in Model-1 to have a better pKi value is sp3N_sp2O_8B. It counts the total number of sp^3^-hybridized nitrogen atoms present within eight bonds of sp^2^-hybridized oxygen atoms. Of the 383 molecules with Ki ≤ 10 nM, 181 molecules lack such a combination of nitrogen and oxygen atoms, whereas the remaining 201 molecules possess only one such combination. In addition, the following pairs of molecules, on comparison, support the observation: 624 with 627 (see Figure 8), 557 with 556 and 555, 271 with 274, 116 with 120, and 117 with 123, to mention a few.

**Figure 9:**
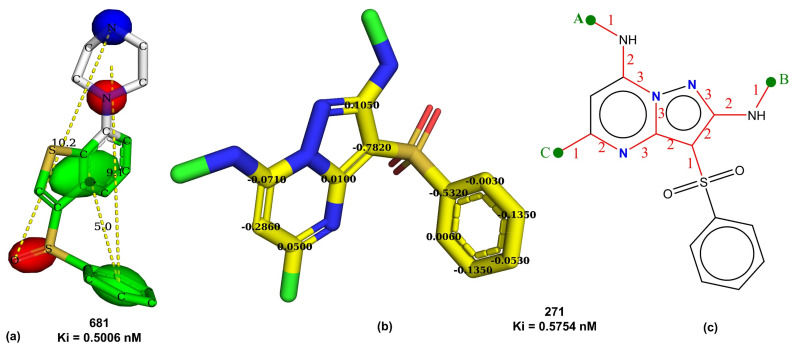
Depiction of (**a**) pharmacophoric feature using molecule 681 (green: hydrophobic, red: H-bond acceptor, blue: H-bond donor regions, dotted lines show distances in Angstrom units), (**b**) yellow atoms represent ringC_S_4Bc in molecule 271, (**c**) fliporingN3B represented by red-colored bonds with numbering using molecule 271.

To summarize the prominent structural features and the pharmacophoric model, we used the two most active molecules, 681 and 271, as representative examples (see Figure 9). The pharmacophoric pattern consists of two hydrophobic regions (green contour), two H-bond acceptor regions (red contour), and one H-bond acceptor region (blue contour), as depicted in Figure 9a using molecule 681. Thus, the pharmacophoric pattern agrees with previous studies [4,12]. Figure 9b depicts the molecular descriptor ringC_S_4Bc in molecule 271. Figure 9c represents the descriptors fliporingN3B and fsp3Cdon1B. The three hydrophobic -CH_3_ groups shown by green dots are responsible for balancing the hydrophilic effect of ring nitrogen atoms, thus enhancing the CNS penetration ability of a molecule. Moreover, the -CH_3_ groups labelled as A and B enhance the value of fsp3Cdon1B, as they are directly attached to donor atoms. Thus, the present work not only captured the pharmacophoric features reported by López-Rodríguez et al. [4,12] but also successfully extended it.

**Figure 10:**
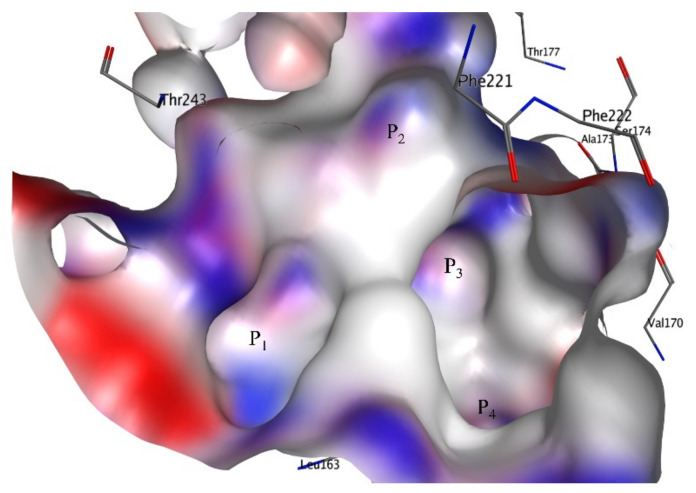
Binding site of 5-HT6 used in the present work.

The molecular docking score spans from −5.7 to −12.2 for molecules in the current dataset (see Supplementary Materials). For the sake of convenience, twenty molecules with the highest and lowest molecular docking scores are tabulated in Table 3. Surprisingly, not only the molecular docking score has a weak correlation of 0.10 with pKi but also many molecules with lower binding affinity values have better docking scores (see Table 3 and Table 4). This could be attributed to the large size of the active site of 5-HT6 (see Figure 10), which allows the adoption of different conformations for molecules. Moreover, recent studies point out that current docking software such as AutoDock, Dock, etc., and respective algorithms for docking scores are inclined toward the flexibility of ligands, which in turn, is associated with the loss of ligand conformational entropy on binding [31]. The molecules with lower binding affinity for 5-HT6 have a high degree of flexibility. Thus, all these factors together resulted in an artificially more favorable binding score for the more flexible decoys than for actives.

## 4. Materials and Methods

In the present study, we have followed the OECD (Organization for Economic Co-operation and Development) guidelines and standard protocol, which has been endorsed by different researchers, for an effective QSAR analysis [15,17,18,21,29,32,33,34,35]. The different steps for developing a model involved a careful selection of a dataset and data curation, followed by 3D structure generation for all molecules, molecular descriptor calculations and their pruning, model development and its thorough validation (internal and external), and mechanistic interpretation [20,36,37]. These steps were executed sequentially to avoid errors and human bias and to ensure the appropriate validation of the model. 

### 4.1. Selection of Dataset

**Figure 11:**
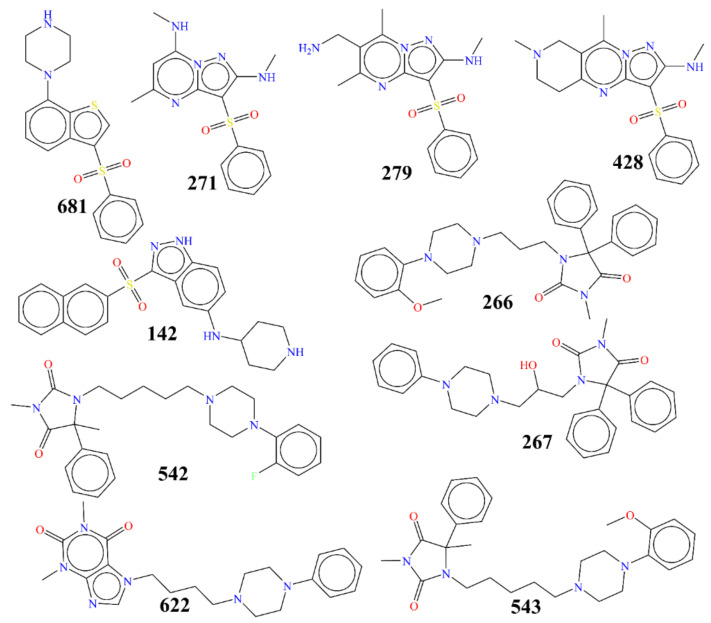
Representative examples from the selected dataset (five most active and five least active molecules) of 5-HT6 ligands.

The size, composition, and structural diversity of the dataset are important characteristics that decide the success and utility of QSAR and molecular docking analysis throughout the pipeline of drug discovery [15,16,17,21,29,32,34,38,39,40,41]. Therefore, a large dataset of 3398 reported ligands for 5-HT6 was downloaded from BindingDB (https://www.bindingdb.org/bind/index.jsp, accessed on 14 January 2022). Then, duplicates, salts, metal derivatives, rule-of-five violators, and molecules with undefined Ki values were removed as a part of data curation [42], which reduced the size of the dataset to only 1278 molecules. The reduced dataset was still diverse enough, with the presence of positional and chain isomers, different heterocyclic and aromatic scaffolds, stereoisomers, etc. The experimental Ki spanned five orders of magnitude (0.5006 nM to 29.07 µM). For a better QSAR analysis, the experimental Ki values were converted to pKi (pKi = −lgKi). To understand the structural variation possessed by the dataset, some highly active and least active molecules are depicted in Figure 11, and their other details are presented in Table 4.

### 4.2. Calculation of Molecular Descriptors and Objective Feature Selection (OFS)

The next step involved the conversion of all SMILES notations to respective 3D-optimized structures using the appropriate method. For this, OpenBabel 3.1 [43] was used to convert SMILES to SDF (structure data file). After that, MOPAC2016 [44] was used to convert SDF to MOL2 using PM3, a semi-empirical method based on the same formalism and equations as the Austin Model-1 (AM1) method, for structure optimization and partial charge assignment. Then, molecular descriptor calculations were accomplished using PyDescriptor [45] and PaDEL [46], which together provided more than 42,000 molecular descriptors for each molecule. Though the myriad numbers of molecular descriptors increase the possibility of achieving a successful QSAR analysis, they also increase the risk of chance correlations or overfitting from noisy redundant descriptors. Therefore, OFS was performed using QSARINS 2.2.4 [47], which eliminated near constant (for 90% molecules) and highly intercorrelated (|R| > 0.90) molecular descriptors. After OFS, the reduced set of molecular descriptors encompassed only 4186 descriptors, which still covered a broad descriptor space due to the presence of 1D to 3D descriptors, fingerprint, and charged-based descriptors as well as atom-pair descriptors. The remaining descriptors were easily interpretable in terms of structural features, thereby increasing the possibility of mechanistic interpretation of the model. 

### 4.3. Splitting the Dataset and Subjective Feature Selection (SFS)

SFS is a crucial step during model building to select a suitable number and set of molecular descriptors using a suitable feature selection algorithm such as stepwise regression, genetic algorithm, etc. For the appropriate training and validation of the model, before actual model building, the dataset was randomly divided into a training set (80%, i.e., 1024 molecules) and a prediction set (20%, i.e., 254 molecules). The random division of a dataset into an 80:20 proportion was performed to avoid any bias and minimize information leakage [32,48], to confirm the external predictive ability of the model, and to achieve a better composition of the training and prediction sets [29,34]. The training set was used only for the selection of the appropriate number and set of molecular descriptors. The prediction set, also termed the external validation set, was used only for the validation of the newly developed model.

**Figure 12:**
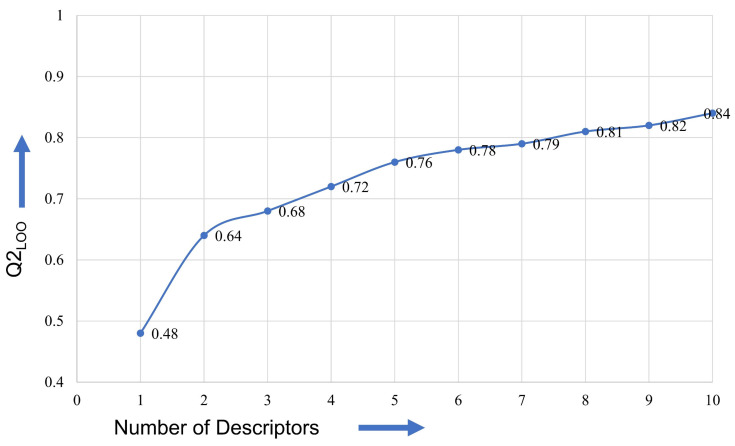
Plot of number of descriptors against leave-one-out coefficient of determination, Q^2^_LOO_, to identify the optimum number of descriptors.

It is essential to include the optimum number of molecular descriptors in the model to avoid over- and underfitting. Consequently, a simple graphical method was used to identify the optimum number of descriptors for a model. Usually, the successive addition of a variable (molecular descriptor) in a multilinear regression (MLR) model augments the value of Q^2^_LOO_ until the final elevation point is achieved [25,29]. After that, there is little or poor augmentation to the value of Q^2^_LOO_. Hence, the number of molecular descriptors matching the elevation point were considered optimum for model building [36]. This is depicted in Figure 12 as a graph. From Figure 12, the final elevation point is matched to six molecular descriptors. Therefore, the heuristic search was confined to including only six molecular descriptors in the QSAR model using a multi-regression analysis. The set of molecular descriptors were selected using a genetic algorithm as a feature selection algorithm and using Q^2^_LOO_ as the fitness parameter along with the genetic algorithm–multilinear regression (GA-MLR) method using QSARINS-2.2.4 [47].

### 4.4. Building Regression Model and Its Validation

The search for six molecular descriptors for the development model ultimately provided different combinations of different molecular descriptors. However, only one combination of molecular descriptors was selected due to the statistical performance and the satisfaction of the following stringent parameters and criteria, which have been suggested by other researchers [16,22,23,24,25,26,27,28,29]:

R^2^_tr_ ≥ 0.6; Q^2^_LOO_ ≥ 0.5; Q^2^_LMO_ (cross-validated coefficient of determination for leave-many-out) ≥ 0.6; R^2^_tr_ > Q^2^_LOO_; R^2^_ex_ ≥ 0.6, CCC (concordance correlation coefficient) ≥ 0.80; Q^2^-F^n^ ≥ 0.60; high values of the external validation parameters R^2^_ex_, Q^2^_F1_, Q^2^_F2_, and Q^2^_F3_; and low values of R^2^_Yscr_ (coefficient of determination for Y-randomization), RMSE (root-mean-square error), and MAE (mean absolute error), with RMSE_tr_ < RMSE_cv_.

The formulae and other details of these statistical parameters are available in the Supplementary Materials. For further validation, it was essential to determine the applicability domain of the QSAR model [49,50]. For this, we used a Williams plot (standardized residuals vs. hat values) to evaluate the applicability domain of the QSAR model [47].

### 4.5. Molecular Docking

Since the X-ray-resolved structure of 5-HT6 is not available, an appropriately validated and reported homology model built using a β2 receptor template (PDB ID: 4LDE) by Vanda et al. [8] was used for molecular docking [6,7] in the present work. 4LDE (selected template) and 5-HT6 (modelled receptor) possess equivalent positions for the most conserved amino acid in each helix and motifs characteristic for class A GPCRs [6,7,8]. The sequences of 5-HT6 and 4LDE were retrieved from the UniProtKB/Swiss-Prot database [8]. The protein was prepared using Chimera (www.cgl.ucsf.edu/chimera/, accessed on 14 January 2022) with default settings. Then, for the execution of molecular docking, AutoDock Vina [51], available in DockingApp [52], which allows users to select flexible residues of the input receptor, was used with flexible docking for virtual screening with default settings. The binding site of 5-HT6, which consists of four major grooves, P1-P4, used in the present work, is depicted in Figure 10. The binding site was used for automated molecular docking for all the molecules. Further details of molecular docking are available in the Supplementary Materials.

## 5. Conclusions

The present work has resulted in the development of a statistically predictive GA-MLR model with a balance of excellent predictive ability (R^2^_tr_ = 0.78, R^2^_ex_ = 0.77) and novel mechanistic interpretations. The present QSAR and molecular docking analysis has successfully identified the importance of steric bulkiness, polar groups, and substitution with respect to the center of mass. It also highlighted the role played by partial charges on ring C atoms present near sulfur atoms. Further, Model-1 successfully identified that a conditional occurrence of lipophilic atoms/groups with respect to nitrogen atoms is better for having a higher pKi value. The crucial role played by the interrelationships between specific types of atoms, such as sp3-hybridized carbon/nitrogen atoms, ring nitrogen atoms, etc., further escalated the expediency of Model-1. The model possesses high external predictive ability, which is evident from its statistical performance. In conclusion, the present work not only effectively identified reported and novel unreported pharmacophoric features associated with 5-HT6 but also offers a highly predictive QSAR model. It could be beneficial throughout the drug discovery pipeline to optimize the lead compounds to develop a better therapeutic for 5-HT6.

## Figures and Tables

**Table 1:** Some details of constituent molecular descriptors present in Model-1.

Molecular Descriptor	Description	Software Used for Calculation	Correlation with pKi
com_Hhyd_3A	Total number of hydrogen atoms with partial charge in the range of ±0.2 within 3 Å from center of mass of molecule	PyDescriptor	−0.625
ringC_S_4Bc	Sum of partial charges on ring carbon atoms present within four bonds from sulfur atom	PyDescriptor	−0.696
flipo&S_ringN3B	Frequency of occurrence of ring nitrogen atom present exactly at three bonds from lipophilic atom	PyDescriptor	−0.248
sp3N_sp2O_8B	Total number of sp3-hybridized nitrogen atoms present within eight bonds from sp2-hybridized oxygen atoms	PyDescriptor	−0.133
KRFPC620	Nitrogen attached to three CH3CH2- groups	PaDEL	−0.444
fsp3Cdon1B	Frequency of occurrence of H-bond donor atom bonded with sp3-hybridized carbon atom	PyDescriptor	0.026

**Table 2:** Selected statistical validation parameters for Model-1.

Parameter	Value	Parameter	Value
R^2^_tr_	0.783	Q^2^_LMO_	0.779
R^2^_adj._	0.781	R^2^_Yscr_	0.006
RMSE_tr_	0.419	RMSE_ex_	0.425
MAE_tr_	0.350	MAE_ex_	0.357
CCC_tr_	0.878	R^2^_ex_	0.772
R^2^_cv_ (Q^2^loo)	0.780	Q^2^-F^1^	0.768
RMSE_cv_	0.422	Q^2^-F^2^	0.768
MAE_cv_	0.352	Q^2^-F^3^	0.777
CCC_cv_	0.876	CCC_ex_	0.871

Note: tr—Training, cv—Cross-validation, ex—External.

**Table 3:** The molecular docking score and Ki (nM) for ten molecules with the highest and lowest molecular docking scores.

Molecule Number	SMILES	Ki (nM)	Affinity- Docking Score (Kcal/mol)
741	CC(=O)Nc(cc1)ccc1CCNICC2Cc(c23)ccc4c3ccn4S(=O)(=O)c5ccccc5	79.43	−12.2
134	C1NCCCC1C(=O)Nc(ccc2)c(c23)[nH]nc3S(=O)(=O)c4cccc(c45)cccc5	9.8	−12
489	c1cccc(c12)[nH]cc2C(C3=O)CC(=O)N3CCCN(CC4)CCC4c5c[nH]c(c56)ccc(c6)OC	264	−11.9
490	c1cccc(c12)[nH]cc2C(C3=O)CC(=O)N3CCN(CC4)CCC4c5c[nH]c(c56)ccc(F)c6	1146	−11.8
1093	CC(=O)Nc(n1)[nH]c(c1C)-c2cn(c(c23)cccc3)S(=O)(=O)c4cccc(c45)cccc5	13	−11.7
668	CI(C)CCC1c2c[nH]c(c23)ccc(c3)NS(=O)(=O)c(ccc4)c(c45)nccc5	21.2	−11.7
133	C1CCCCN1CCC(=O)Nc(ccc2)c(c23)[nH]nc3S(=O)(=O)c4cccc(c45)cccc5	24	−11.7
381	FC(F)(F)c1cc(ccc1)S(=O)(=O)n(c(c2c34)CCC(C2)N)c3ccc(c4)OC	39.1	−11.7
805	c1cccc(c12)ccc(c2)S(=O)(=O)NCCN(CC3)CC=C3c4c[nH]c(c45)ccc(F)c5	67	−11.7
628	c1cccc(c12)CN([C@@H](C2)C(=O)N)C(=O)CCCCN3CCN(CC3)c(cccc4)c4-c5ccccc5	594	−11.7
1086	CCn1cncc1-c2c[nH]c(c23)ccc(Br)c3	1349	−7.3
1016	CCn1cncc1-c2c[nH]c(c23)cccc3	3020	−7.3
1203	N1CCC[C@@H]1Cc2c[nH]c(c23)cccc3	60	−7.2
1202	c1cccc(c12)n(cc2)C[C@H]3CCCN3C	550	−7.2
339	NCCc1cc(ccc1)Sc2ccccc2	115	−7.1
93	CCN(CC)CCc1c[nH]c(c12)cccc2	575	−6.4
738	NCCc1c[nH]c(c12)ccc(c2)O	42.333	−6.2
534	CC(N)Cc1c[nH]c(c12)cccc2	910.505	−6.2
444	NCCc1c[nH]c(c12)ccnc2	64	−5.7
445	CN(C)CCc1c[nH]c(c12)ccnc2	100	−5.7

**Table 4:** SMILES (simplified molecular input line entry system) notation, Ki (nM), pKi (M), and molecular docking score for the five most and five least active molecules of the selected dataset.

Molecule Number	SMILES (Simplified Molecular Input Line Entry System) Notation	Ki(nM)	pKi(M)	Docking Score (Kcal/mol)
681	C1CNCCN1c(ccc2)c(c23)scc3S(=O)(=O)c4ccccc4	0.5006	9.301	−9
271	Cc(n1)cc(NC)n(c12)nc(NC)c2S(=O)(=O)c3ccccc3	0.5754	9.24	−8.3
142	C1CNCCC1Nc(c2)ccc(c23)[nH]nc3S(=O)(=O)c(c4)ccc(c45)cccc5	0.6	9.222	−10.8
279	Cc(n1)IN)c(C)n(c12)nc(NC)c2S(=O)(=O)c3ccccc3	0.6457	9.19	−8.4
428	CN(C1)CIn2)c1c(C)n(c23)nc(NC)c3S(=O)(=O)c4ccccc4	0.66	9.18	−9.1
622	O=I(C)c(=O)n(C)c(c12)ncn2CCCCN3CCN(CC3)c4ccccc4	14210	4.847	−9.6
543	c1cccc(OC)c1N(CC2)CCICCCCI(=O)N(C)C(=O)C3(C)c4ccccc4	14650	4.834	−10.1
267	c1ccccc1N(CICCN2CC(O)CN3C(=O)N(C)C(=O)C3(c4ccccc4)c5ccccc5	20410	4.69	−9.7
542	c1cccc(F)cICC2)I2CCCCCN3C(=O)N(C)C(=O)C3(C)c4ccccc4	25520	4.593	−10.1
266	c1cI(OC)c1N(CC2)CCN2CCCN3C(=O)N(C)C(=O)C3(c4ccccc4)c5ccccc5	29070	4.537	−10.8

## Data Availability

The data is contained within the article and Supplementary Materials.

## Supplementary Materials

The following supporting information can be downloaded at: https://www.mdpi.com/article/10.3390/ph15070834/s1: Additional statistical parameters for model-1, Additional details of experimental procedure for molecular docking, Statistical symbols with names and explanations, Molecular descriptors for all molecules used in model-1, their correlation matrix and molecular docking score.

## Abbreviations

SMILES	Simplified molecular-input line-entry system
GA	Genetic algorithm
MLR	Multiple linear regression
QSAR	Quantitative structure−activity relationship
WHO	World Health Organization
ADMET	Absorption, distribution, metabolism, excretion, and toxicity
OLS	Ordinary least square
QSARINS	QSAR Insubria
OECD	Organisation for Economic Co-operation and Development
