# Supplementary material for: Perceiving the Concealed and Unreported Pharmacophoric Features of the 5-Hydroxytryptamine Receptor Using Balanced QSAR Analysis
Source: Pharmaceuticals (Basel). 2022 Jul 5;15(7):834. doi: 10.3390/ph15070834 (PMC9316833; doi:10.3390/ph15070834)
Supplement: Supplementary file 1 [file pharmaceuticals-15-00834-s001.zip › Supplementary Material.pdf]

Perceiving the Concealed and Unreported Pharmacophoric Features of the 5-hydroxytryptamine Receptor Using Balanced QSAR Analysis

Syed Nasir Abbas Bukhari [1,*], Mervat Abdelaziz Elsherif [2], Kashaf Junaid [3], Hasan Ejaz [3], Pravej Alam [4], Abdul Samad [5], Rahul D. Jawarkar [6] and Vijay H. Masand [7,*]

1 Department of Pharmaceutical Chemistry, College of Pharmacy, Jouf University, Sakaka 72388, Saudi Arabia
2 Chemistry Department, College of Science, Jouf University, Sakaka 72388, Saudi Arabia; maelsherif@ju.edu.sa
3 Department of Clinical Laboratory Sciences, College of Applied Medical Sciences, Jouf University,
Sakaka 72388, Saudi Arabia; kjunaid@ju.edu.sa (K.J.); hetariq@ju.edu.sa (H.E.)
4 Department of Biology, College of Science and Humanities, Prince Sattam Bin Abdulaziz University,
Al-Kharj 11942, Saudi Arabia; alamprez@gmail.com
5 Department of Pharmaceutical Chemistry, Faculty of Pharmacy, Tishk International University,
Erbil 44001, Iraq; abdul.samad@tiu.edu.iq
6 Department of Medicinal Chemistry, Dr. Rajendra Gode Institute of Pharmacy, University-Mardi Road, Amravati 444603, Maharashtra, India; rahuljawarkar@gmail.com
7 Department of Chemistry, Vidya Bharati Mahavidyalaya, Amravati 444602, Maharashtra, India
* Correspondence: sbukhari@ju.edu.sa (S.N.A.B.); vijaymasand@gmail.com (V.H.M.)

Statistical validation parameters for model-1:

R2tr = 0.783, R2adj. = 0.781, LOF = 0.18, Kxx = 0.39, ΔK = 0.071, RMSEtr = 0.419, MAEtr = 0.35, RSStr = 179.941, CCCtr = 0.878, s = 0.421, F = 609.878, R2cv (Q2loo) = 0.78, RMSEcv = 0.422, MAEcv = 0.352, PRESScv = 182.45, CCCcv = 0.876, Q2LMO = 0.779, R2Yscr = 0.006, Q2Yscr = -0.008, RMSEex = 0.425, MAEex = 0.357, PRESSext = 45.78, R2ex = 0.772, Q2-F1 = 0.768, Q2-F2 = 0.768, Q2-F3 = 0.777, CCCex = 0.871, R2-ExPy = 0.772, R'o2 = 0.711, k' = 1.005, 1-(R2/ R'o2) = 0.079, r'2m = 0.581, Ro2 = 0.772, k = 0.992, 1-(R2-ExPy/ Ro2) = 0, r2m = 0.766

Experimental procedure- Molecular docking:

For molecular docking, the protein preparation was accomplished using DockPrep protocol available in Chimera. It involved removal of water/solvent molecules, fixing of non-standard residues, adding hydrogens and charges (AMBER force field). Then, DockingApp, which uses AutoDock vina for actual docking and docking score calculations, was used for flexible molecular

docking of all the molecules inside the active site. The 'Grid Type' and 'VS Type' were set to 'Select grid-bounding residues' and 'Flexible Docking' respectively. The residues constituting the four major grooves P1-P4 were set as flexible residues. The structure of protein and all ligands were provided in '.pdb' format. The 'number of CPU cores to use' was set to 20 to speed-up the virtual screening process.

Statistical symbols with names and explanations:

R^2 – correlation coefficient, Q^2 – leave-one-out 'crossvalidated R^2', R^2_{adj} - adjusted R^2, SEE – standard error of estimates, RMSE - root mean squared error, MAE - mean absolute error, CCC - concordance correlation coefficient, for the training (tr), and test (ex) sets; R^2_{LMO} and Q^2_{LMO} – leave many-out correlation coefficient and cross-validation coefficients; R^2_{Yrand} and Q^2_{Yrand} – Y- scramble correlation and cross-validation coefficients;

Statistical parameters for used for validation of QSAR models:

$$R^2 = 1 - \frac{\sum(y_i - \hat{y}_i)^2}{\sum(y_i - \bar{y})^2}$$

where y_i are the observed values of the response, ȳ the corresponding average, ŷ are the calculated values

$$Q^2 = 1 - \frac{\sum(y_i - \hat{y}_i)^2}{\sum(y_i - \bar{y})^2}$$

where y_i are the observed values of the response, ȳ the corresponding average, ŷ are the values predicted for each object when it is not in the training set.

$$Q^2_{F1} = 1 - \frac{\sum_{i=1}^{n_{EXT}}(y_i - \hat{y}_i)^2}{\sum_{i=1}^{n_{EXT}}(y_i - \bar{y}_{TR})^2}$$

where y_i are the observed values of the response, ȳ the corresponding average, ŷ are the calculated values

$$Q^2_{F2} = 1 - \frac{\sum_{i=1}^{n_{EXT}}(y_i - \hat{y}_i)^2}{\sum_{i=1}^{n_{EXT}}(y_i - \bar{y}_{EXT})^2}$$

where y_i are the observed values of the response, $\bar{y}$ the corresponding average, $\hat{y}$ are the calculated values

$$Q_{F3}^2 = 1 - \frac{\left[\sum_{i=1}^{n_{EXT}}(y_i - \hat{y}_i)^2\right]/n_{EXT}}{\left[\sum_{i=1}^{n_{TR}}(y_i - \bar{y}_{TR})^2\right]/n_{TR}}$$

where y_i are the observed values of the response, $\bar{y}$ the corresponding average, $\hat{y}$ are the calculated values

$$CCC = \frac{2\sum_{i=1}^{n_{EXT}}(y_i - \bar{y})(\hat{y}_i - \bar{\hat{y}})}{\sum_{i=1}^{n_{EXT}}(y_i - \bar{y})^2 + \sum_{i=1}^{n_{EXT}}(\hat{y}_i - \bar{\hat{y}})^2 + n_{EXT}(\bar{y} - \bar{\hat{y}})^2}$$

$$k = \frac{\sum_{i=i}^{n_{EXT}} y_i\hat{y}_i}{\sum_{i=1}^{n_{EXT}} \hat{y}_i^2}$$

$$k' = \frac{\sum_{i=i}^{n_{EXT}} y_i\hat{y}_i}{\sum_{i=1}^{n_{EXT}} y_i^2}$$

$$r_m^2 = r^2\left(1 - \sqrt{r^2 - r_0^2}\right)$$

$$\overline{r_m^2} = \frac{(r_m^2 + r_m'^2)}{2}$$

$$RMSE = \sqrt{\frac{\sum_{i=1}^{n_{EXT}}(y_i - \hat{y}_i)^2}{n_{EXT}}}$$

$$MAE = \frac{\sum_{i=1}^{n_{EXT}}|y_i - \hat{y}_i|}{n_{EXT}}$$

where y_i are the observed values of the response, $\bar{y}$ the corresponding average, $\hat{y}$ are the calculated values
